# Effects of dance for Parkinson’s disease on habitual physical activity: a quasi-experimental controlled study

**DOI:** 10.3389/fnhum.2026.1962191

**Published:** 2026-10-13

**Authors:** Hewa H. N. Kalyani, Karen A. Sullivan, Gene M. Moyle, Sandra G. Brauer, Erica R. Jeffrey, Graham K. Kerr

**Affiliations:** 1 School of Health, Psychological and Medical Sciences, University of Southern Queensland, Toowoomba, QLD, Australia; 2 Movement Neuroscience Group, School of Exercise and Nutrition Sciences, Faculty of Health, Queensland University of Technology, Brisbane, QLD, Australia; 3 School of Psychology and Counselling, Faculty of Health, Queensland University of Technology, Brisbane, QLD, Australia; 4 School of Creative Practice, Faculty of Creative Industries, Queensland University of Technology, Brisbane, QLD, Australia; 5 School of Health and Rehabilitation Sciences, Faculty of Health, Medicine and Behavioural Sciences, University of Queensland, Brisbane, QLD, Australia; 6 Queensland Ballet, South Brisbane, QLD, Australia; 7 Dance for Parkinson's Australia, Brisbane, QLD, Australia

**Keywords:** dance, functional mobility, habitual physical activity, Parkinson’s disease, rehabilitation

## Abstract

**Background:**

Despite symptomatic therapies for PD, habitual physical activity inevitably declines as the disease progresses, leading to increased sedentary behavior. While dance programs have demonstrated a multitude of benefits including motor, non-motor, and psychological aspects, it has not yet been objectively determined if dance can improve habitual physical activity in people living with PD.

**Aim:**

This study explored whether participation in Dance for Parkinson’s Disease classes was associated with changes in habitual physical activity in people with PD.

**Design:**

A quasi-experimental controlled efficacy study, with pre and post-testing of two parallel groups (dance versus control).

**Population:**

Thirty-three participants with PD allocated to a dance group (DG; *n* = 17; age = 65.8 ± 11.7 years) or control group (CG: *n* = 16; age = 67.0 ± 7.7 years). Participants were cognitively intact (Addenbrooke’s score: DG = 93.2 ± 3.6, CG = 92.6 ± 4.3) and in early stage PD (Hoehn & Yahr: DG = 1.6 ± 0.7, CG = 1.5 ± 0.8).

**Methods:**

The DG undertook a one-hour Dance for Parkinson’s Disease (DfPD®) -based class, twice weekly for 12 weeks (intervention). The CG had treatment as usual. ActivPAL™ accelerometers monitored habitual physical activity for five consecutive days pre-and post-intervention. Sit/lie duration (min), standing duration (min), stepping duration (min), sit/stand movements (count), stand/sit movements (count), daily step count, and energy expenditure (MET-h/day) were measured.

**Results:**

No significant between-group differences were observed for any ActivPAL outcome. However, increases in daily stepping duration and step count demonstrated moderate-to-large effect sizes (*d* = 0.72 and *d* = 0.91, respectively).

**Conclusion:**

Participation in a dance for Parkinson’s disease program did not significantly alter pre- to post-program habitual physical activity. Larger adequately powered trials are required to determine whether participation in dance programs can influence habitual physical activity in people with Parkinson’s disease.

## 1 Introduction

People with PD engage in approximately 30% less habitual physical activity compared to healthy age-matched controls (Lord et al., 2013; van Nimwegen et al., 2011). This phenomenon is demonstrated across disease severity. For example, people with mild PD have substantially reduced levels of ambulatory activity (Lord et al., 2013). People with mild to moderate PD spend more time per day in sedentary versus physically active behaviors (Benka Wallén et al., 2015). These changes in the amount of physical activity by people with PD, are also evident when examining activity patterns. Compared with age- and gender-matched controls, people with PD have longer-duration sedentary bouts that are less frequently interrupted by upright activity (Chastin et al., 2010). Addressing these changes in physical activity is a key priority for people living with PD.

The reduction of habitual physical activity in people with PD represents an important clinical risk to overall health and functioning. Physical activity has an important neuroprotective effect in early stage PD, helping to normalize cortical excitability (Fisher et al., 2008; Zigmond and Smeyne, 2014). Decreased habitual physical activity in PD increases the risk for comorbidities including cardiovascular disease, diabetes mellitus, hypertension, cognitive impairment, osteoporosis, and depression (Warburton et al., 2006). For example, a longitudinal study showed that the decline in physical activity in 301 people with PD significantly increased their risk of mild cognitive impairment and dementia. Such findings suggest the possibility of reducing this risk through increased activity, viewing it as a “modifiable risk factor” (Jones et al., 2022).

Current World Health Organization guidelines recommend that older adults and people living with chronic conditions accumulate 150–300 min of moderate-intensity physical activity per week, engage in multicomponent activities that include balance and functional training, and minimize sedentary behavior (World Health Organization, 2020). Similarly, the American College of Sports Medicine guidelines for people with PD recommend regular aerobic, strength, balance, and functional exercise distributed throughout the week to optimize health, mobility, and participation (Ozemek et al., 2025). Dance aligns closely with these recommendations because it combines aerobic activity, balance challenge, motor skill practice, cognitive engagement, and social participation within a single intervention.

Understanding the reasons for decreased habitual activity in PD could be key to finding solutions to improve it. Disabling motor symptoms, such as gait and balance problems, as well as diverse non-motor problems including cognitive decline, apathy, and depression, potentially contribute to the reduced physical activity (van Nimwegen et al., 2011). Personal and social factors can negatively impact physical activity (Lamont et al., 2016); for example, through reduced availability of social support or supervision (Foster et al., 2005; Schootemeijer et al., 2020). In addition, individuals with PD may consciously restrict their activity because they perceive it as necessary to reduce their risk of falling, which can further contribute to lower habitual activity levels (Pickering et al., 2007). Long-term exercise participation may also be facilitated by education, social support, feedback, and professional guidance (Schootemeijer et al., 2020). Consequently, improvements in physical function alone may not necessarily lead to increases in habitual physical activity.

Despite the many factors that may interact to reduce physical activity in people with PD, it can be significantly increased through structured exercise / activity programs. There is now substantial evidence that PD-targeted dance programs bring a multitude of benefits including improved gait, balance, cognition, anxiety, depression, and quality of life (Andreasson et al., 2024; Bearss and DeSouza, 2021; Bek et al., 2022; DeFarias et al., 2026; Haputhanthirige et al., 2023; Kalyani et al., 2020; Kalyani et al., 2019b; Hasan et al., 2022), potentially addressing some of the factors that reduce habitual activity. Specifically, dance for Parkinson’s disease programs have demonstrated beneficial effects on functional mobility and quality of life (Haputhanthirige et al., 2023; Kalyani et al., 2020; Kalyani et al., 2019b). Dance also incorporates physical, cognitive, sensory, and social components that may facilitate participation in regular physical activity and reduce sedentary lifestyles. These benefits may arise from the features commonly incorporated in dance for Parkinson’s disease programs, which is not a dance genre/style per se, but a framework for the use of dance styles within an individually tailored, graduated program, incorporating social and group elements. The improvements seen in dance for Parkinson’s disease programs have the potential to generalize, including increasing participants’ post-program habitual physical activity levels.

To our knowledge, no previous study has evaluated habitual physical activity following a dance for Parkinson’s disease intervention using objective monitoring of physical activity. Such an investigation is important because program-based improvements in physical function ideally also increase habitual physical activity. We hypothesized that participation in a 12-week dance for Parkinson’s disease program would result in greater improvements in objectively measured habitual physical activity in a dance group compared to a treatment-as-usual (control) group of PD participants.

## 2 Materials and methods

This prospective quasi-experimental controlled study had a quasi-experimental parallel-group pre- versus post-test design and was carried out at the Institute of Health and Biomedical Innovation, Queensland University of Technology (QUT). Participants gave written informed consent in accordance with the Declaration of Helsinki (World Medical Association, 2013) and the experimental protocol was approved by the Human Research Ethics Committee at QUT (#1700000005). The study was registered in the Australian New Zealand Clinical Trials Registry (ANZCTR) (#ACTRN12618001834246). The recruitment was initiated in March 2017. Unblinded assessments commenced in July 2017 (baseline) with follow-ups completed in March 2018.

### 2.1 Participants

Participants were recruited from PD support groups in Queensland, advertising on the Parkinson’s Queensland website, distributing flyers to participants in the existing dance for Parkinson’s disease class at Queensland Ballet (QB), through the “Radio Parkies” radio show, and via the QUT email system. Forty-nine people with PD expressed interest in the study and were interviewed by phone to determine eligibility. Eligible participants: (1) required a clinical diagnosis of idiopathic PD, using the published diagnostic criteria for clinically defined *definite PD* (Racette et al., 1999); (2) were aged between 40 and 85 years; (3) had mild to moderate stage disease (Hoehn and Yahr stages I-III) (Goetz et al., 2004); (4) were free from dementia (Addenbrooke’s Cognitive Examination: ACE > 82) (Goetz et al., 2004); (5) had no medical, neurological (other than PD), musculoskeletal, cardiovascular or respiratory abnormalities; and (6) could walk independently for ≥ 3 m without an assistive device. Participants were on stable medication regimens and were tested in the “ON” medication phase (within 3 hours post-PD medication).

Following phone screening, 38 people were eligible for participation. Group allocation was pseudo-random (dance group: DG = 19 and control group: CG = 19) to accommodate issues such as logistical constraints (e.g., participant availability). During baseline assessment, two DG participants and three CG participants discontinued, leaving 17 and 16 participants in the DG and CG, respectively (Figure 1).

**Figure 1:**
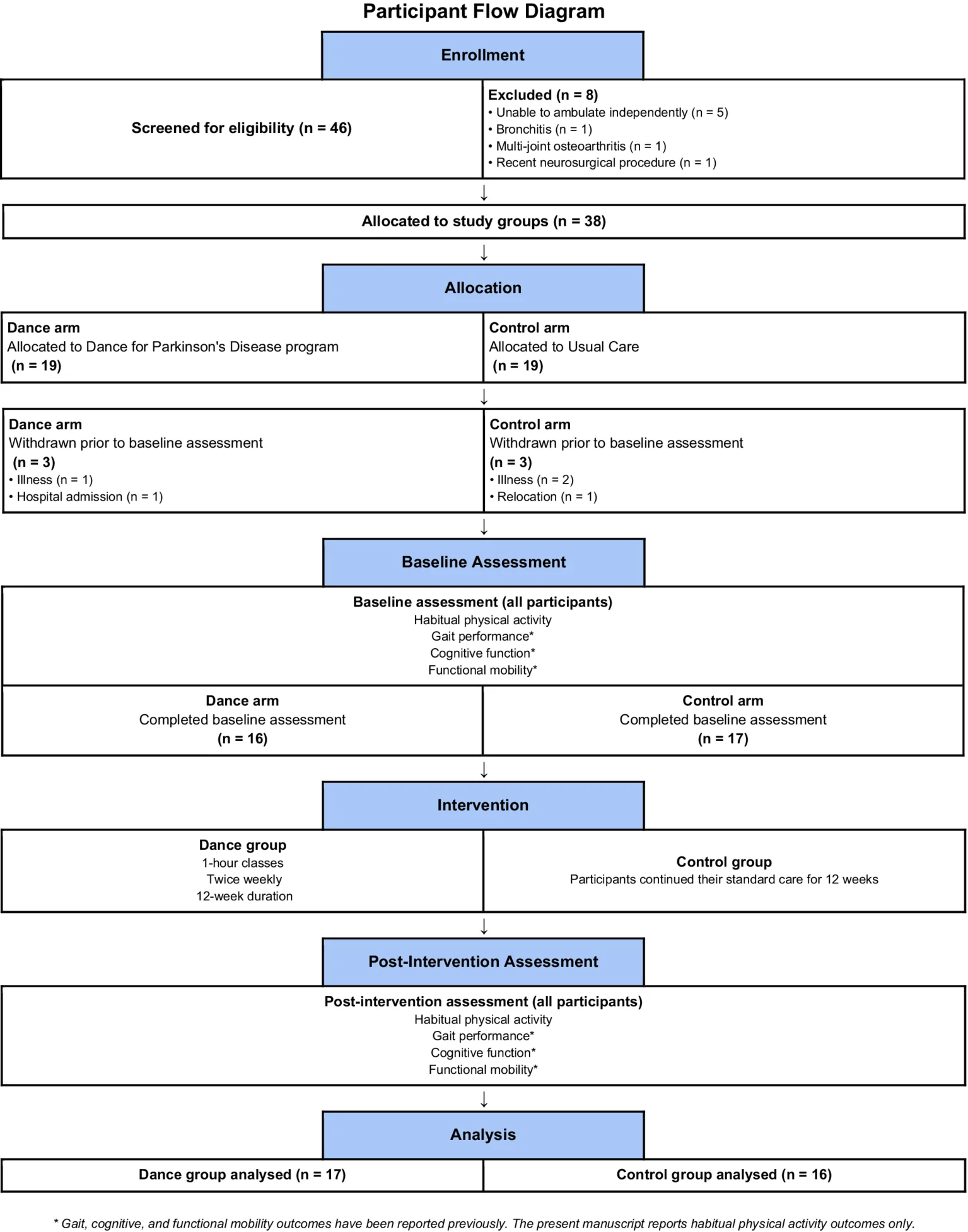
Consort diagram of participant recruitment, group allocation and tracking over the course of the study.

### 2.2 Intervention

The intervention was a dance for Parkinson’s disease program based on the Dance for Parkinson’s Disease (DfPD®) approach (Westheimer, 2008), and delivered by DfPD®-trained instructors from Queensland Ballet. The dance classes commenced in August 2017 and continued for 3 months as a one-hour twice-weekly class. Each class comprised 30 min of seated dance, followed by approximately 15 min of supported standing dance and 15 min of standing dance involving movement across the floor. The program incorporated elements of ballet, modern dance, jazz, tap, choreographic repertory, and limited Scottish dance. Recorded music ranging from classical to contemporary styles was used throughout the classes. Progression occurred through increasing movement complexity, coordination demands, pacing, memory challenges, improvisation, and sequence variation rather than changes to class duration. While the overall class structure and duration of the seated, supported standing, and movement across the floor components remained consistent throughout the 12-week program, progression was implemented systematically at the group level through increasingly challenging movement tasks. Participants were encouraged to perform activities at a level appropriate to their individual capabilities, allowing adaptations where required. The intervention was added to each participant’s usual PD care.

The dance classes incorporated several key components of the DfPD® methodology. These classes used different attentional cues such as auditory, visual, and somatosensory, which may facilitate movement through the recruitment of alternative neural pathways (Rubinstein et al., 2002). Music provided an auditory cue to facilitate movement and support movement initiation and coordination among participants (Batson et al., 2016). Visual feedback was obtained through observation of the dance instructors. Participants received sensory input when holding hands with partners in a circle or line. This light touch acts as a somatosensory cue to focus attention on balance (Westheimer et al., 2015). Somatosensory input provided through partner-based activities may further enhance postural control and balance. Among the various cognitive strategies used in these dance classes, internal imagery was used to encourage participants to mentally rehearse and coordinate movements (Westheimer, 2008). The class had some aspects of improvisation, requiring participants to generate spontaneous movement sequences and increasing cognitive engagement (Batson et al., 2016). Repetition, variations on familiar sequences, and new sequences were also incorporated (Westheimer, 2008). Variations in movement speed, quality, and sequence complexity were used to challenge motor control, attention, and memory (Westheimer et al., 2015). Collectively, these strategies provided progressive mental and physical challenges while promoting motor learning and movement control.

### 2.3 Assessment protocol and activity measurement

Participants attended four assessments, 3 hours each: two at the baseline and two post-intervention. An assessment battery was administered to measure various outcomes including disease severity (Table 1). The current manuscript represents a secondary analysis of a broader study cohort that has previously generated publications examining gait, dual-task gait, cognition, functional mobility, psychological symptoms, and quality of life (Haputhanthirige et al., 2023; Kalyani et al., 2020; Kalyani et al., 2019a). The present analysis is novel because it specifically examines objectively measured habitual physical activity using ActivPAL™ monitoring, outcomes that have not been previously reported.

**Table 1:** Descriptive statistics and group comparisons of baseline and PD characteristics.

Characteristics	Dance group (*n* = 17)	Control group (*n* = 16)	*p*-value
*Gender, n (%)*			
Male	3 (17.6%)	10 (62.5%)	
Female	14 (82.4%)	6 (37.5%)	**0.008**
Age (years), M(SD)	65.24 ± 11.88	66.50 ± 7.70	0.721
Education, *n* (%)			
Primary school	0 (0%)	0 (0%)	
Secondary school	5 (29.4%)	8 (50%)	
Technical college	1 (5.9%)	3 (18.8%)	
University	11 (64.7%)	5 (31.3%)	0.141
Number of falls during previous 6 months, M(SD)	0.18 ± 0.39	0.13 ± 0.34	0.692
Years since PD diagnosis, M(SD)	3.76 ± 2.88	5.94 ± 3.61	0.064
Levodopa equivalent dose (mg/day), M(SD)	555.57 ± 332.41	715.56 ± 418.38	0.261
MDS-UPDRS, M(SD)	73.82 ± 25.67	58.06 ± 22.72	0.072
MDS-UPDRS motor subscale III, M(SD)	38.71 ± 17.67	30.25 ± 15.64	0.157
Hoehn and Yahr scale, M(SD)	1.65 ± 0.79	1.56 ± 0.81	0.763
Addenbrooke’s examination, M(SD)	93.71 ± 3.18	92.69 ± 4.33	0.445

*N* = 33, M, mean; SD, standard deviation; Comparisons are performed with either independent samples *t* test or Pearson Chi-Square test whenever appropriate. MDS-UPDRS: Movement Disorder Society: Unified Parkinson’s Disease Rating Scale. Bold values are significant (i.e., *p*-value < 0.05).

To objectively monitor habitual physical activity, all participants wore ActivPAL™ monitors for five consecutive days at baseline and post-intervention. ActivPAL™ monitors were selected as an established methodology, given prior successful application in studies of people with PD (Mahendran et al., 2016). At baseline assessment, ActivPAL™ monitors were fitted to the midline of the anterior aspect of the thigh using a waterproof, hypoallergenic adhesive dressing (Opsite Flexifix). This placement was selected in accordance with established ActivPAL™ protocols to optimize the identification of sitting/lying, standing, and stepping postures (Mahendran et al., 2016). Participants were advised to wear the ActivPAL™ continuously, including during bathing or showering, and to maintain their usual daily activities. They were asked not to do anything different from what they would usually do while wearing the device. In case of irritation, redness, pain, or itching at the fitting site participants were advised to remove the ActivPAL™ immediately or contact investigators. If the device fell off during the monitoring period, participants were requested to contact the investigators. ActivPAL™ monitors were returned at the subsequent assessment visit for data download and processing.

ActivPAL™ devices passively recorded the following data: sit/lie duration (min), standing duration (min), stepping duration (min), sit-to-stand transitions (count), stand-to-sit transitions (count), daily step count, and energy expenditure (MET-h/day). Volume variables, which were expressed as average daily values over the 5-day monitoring period, were explored (Rochester et al., 2012). Exported data were entered into an Excel spreadsheet to extract the relevant variables. Further processing of the data produced the posture of the participant as either sitting/lying, standing, or stepping. The number of transitions (sit-to-stand and stand-to-sit) was recorded by the device (Grant et al., 2006). Energy expenditure was obtained from the ActivPAL™ software output and expressed as metabolic equivalent hours per day (MET-h/day), representing the cumulative metabolic cost of activity over the monitoring period.

### 2.4 Statistical analysis

Participants who attended at least 80% of the scheduled dance classes were included in the analysis. Complete ActivPAL™ data were available for 16 participants in each group. Descriptive statistics (e.g., means and standard deviations) were calculated for sample characterization and ActivPAL metrics. Participant characteristics at baseline were compared between groups using chi-square tests for categorical variables and independent samples t-tests for continuous variables.

To demonstrate habitual physical activity data patterns across a 24-h period, two cases were selected (mild PD versus moderate PD) for data visualization and illustrative purposes. To examine changes associated with participation in the dance program, linear mixed model analysis was performed on pre-post change scores for each activity parameter. Group allocation was treated as a fixed factor, with participant gender and disease severity included as covariates because of baseline differences and their potential influence on habitual physical activity. Change scores, 95% confidence intervals, *p*-values, and effect sizes were calculated. Data from the five-day monitoring periods were entered separately rather than averaged to account for day-to-day variability in activity patterns. Linear mixed models were estimated using restricted maximum likelihood (REML), with participant entered as a random intercept effect (subject = Index_no) to account for clustering of repeated daily observations within individuals. A variance components covariance structure was specified for the random effects. No additional repeated-measures covariance structure was specified for monitoring day. All statistical procedures were conducted using SPSS version 16, with statistical significance set at *p* < 0.05. Cohen’s d were interpreted according to recommended standards (small = 0.2, medium = 0.5, large = 0.8) (Sullivan and Feinn, 2012).

## 3 Results

The two groups were equivalent in the number of falls during the last 6 months, levodopa equivalent dose, disease severity, and level of cognition (Table 1). Both groups had equivalent education (Secondary School: DG = 5, CG = 8; Tertiary Education: DG = 12, CG = 8). However, the DG had significantly more females than the CG (82.4% versus 37.5%, *p* = 0.008). Because gender differed between groups at baseline, subsequent analyses were adjusted accordingly. The DG had a 92.89% attendance rate, with participants completing at least 20 classes. Apart from the one lost ActivPAL™, no other device-related incidents were reported.

The 24-h habitual physical activity distribution of two selected participants is given in Figures 2, 3. Figure 2 relates to a participant in the mild stage of the disease (Hoehn and Yahr I). Figure 3 relates to a participant in the moderate stage of the disease (Hoehn and Yahr III). Yellow color indicates the sitting/lying period, green indicates the standing phase and red indicates the stepping period. These figures were included for illustrative purposes and demonstrate variation in habitual activity patterns and sedentary behavior across different levels of disease severity.

**Figure 2:**
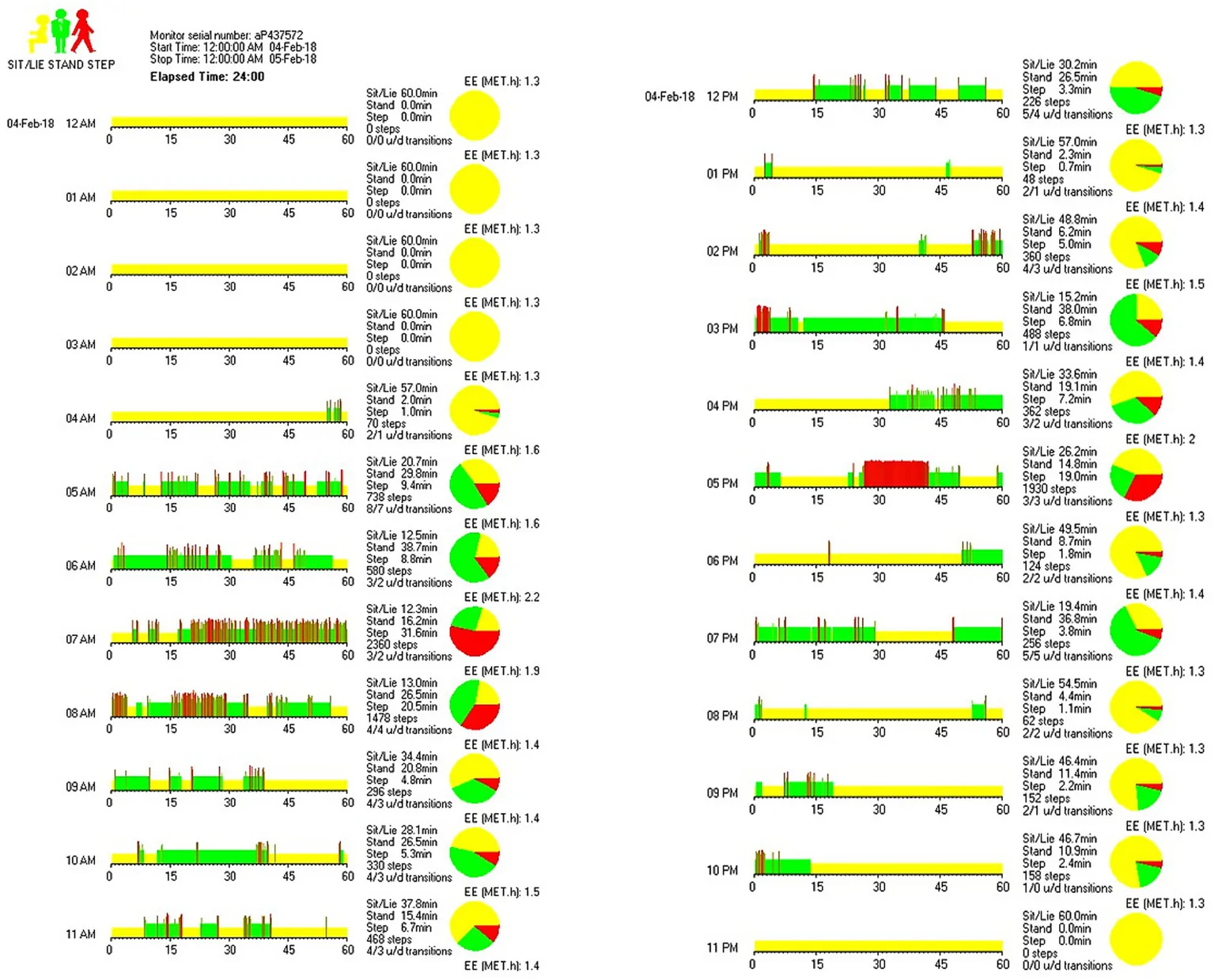
Daily (24 h) activity distribution of a participant in the mild stage of the disease (Modified Hoehn and Yahr I).

**Figure 3:**
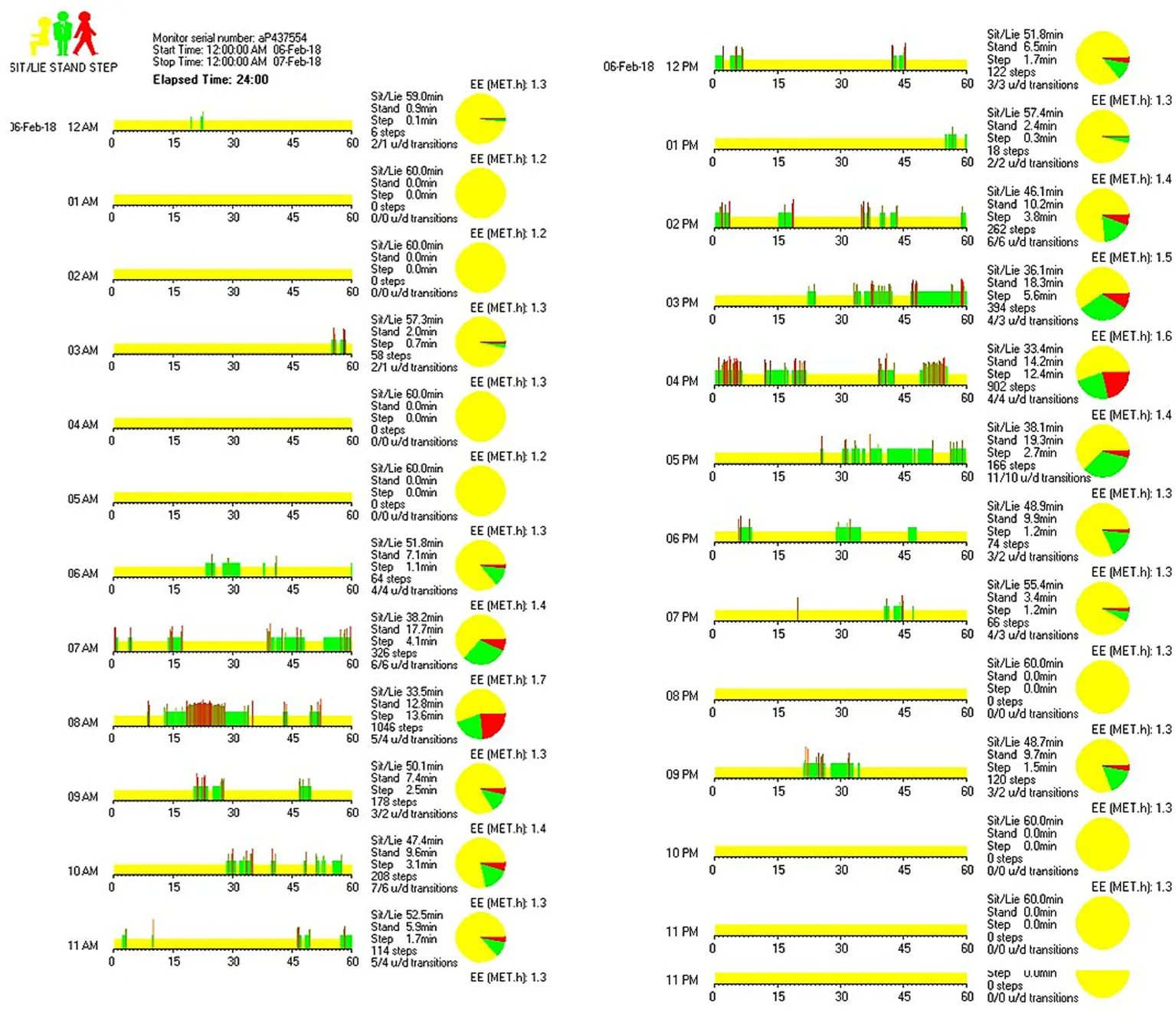
Daily (24 h) activity distribution of a participant in the moderate stage of the disease (Modified Hoehn and Yahr III).

Pre- and post-intervention data collected over 5 days for each participant were further analyzed to determine average daily activity patterns. The results for sit/lie duration, standing duration, stepping duration, sit-to-stand transitions, stand-to-sit transitions, daily step count, and energy expenditure are presented in Table 2. Descriptive statistics showed that daily step count increased in the DG but decreased in the CG over the study period. Similarly, stepping duration increased in the DG and remained largely unchanged in the CG.

**Table 2:** Level of activity measured using ActivPAL™ activity monitors: before and after the 3 months dance intervention.

Walking conditions	Pre-test Mean ± S. D		Post-test Mean ± S. D		Change scores Mean^4^ [95% CI]		*p*-value^6^	Effect size^5^
	Dance (*n* = 16)	Control (*n* = 16)	Dance (*n* = 16)	Control (*n* = 16)	Dance (*n* = 16)	Control (*n* = 16)		
Sit/Lie duration (min)	1150.78 (52.79)	1024.92 (48.13)	1106.72 (52.79)	1029.95 (48.93)	−53.13 [−112.12, 5.85]	3.30 [−49.50, 56.09]	*p* = 0.20	*d* = 0.53
Standing duration (min)	273.81 (45.57)	314.55 (40.76)	306.13 (45.57)	314.30 (40.95)	24.99 [−25.59, 75.56]	9.23 [−35.95, 54.41]	*p* = 0.67	*d* = 0.17
Stepping duration (min)	103.03 (11.12)	94.11 (10.00)	115.00 (11.12)	95.36 (10.07)	18.05 [3.05, 33.05]	−0.84 [−14.34, 12.66]	*p* = 0.10	*d* = 0.72
Sit/stand movement	57.33 (4.77)	52.06 (4.28)	60.25 (4.77)	54.29 (4.32)	5.20 [−1.24, 11.65]	1.09 [−4.70, 6.87]	*p* = 0.39	*d* = 0.35
Stand/sit movements	46.00 (4.51)	41.54 (4.05)	49.21 (4.51)	43.30 (4.08)	5.96 [−0.90, 12.84]	0.55 [−5.61, 6.70]	*p* = 0.29	*d* = 0.44
Number of steps	8354.26 (953.47)	7554.03 (858.01)	9,086 (953.47)	7532.96 (865.55)	1373.92 [118.18, 2829.66]	−2.88 [−1421.15, 844.95]	*p* = 0.08	*d* = 0.91
Energy expenditure (MET.h)	35.82 (1.76)	33.59 (1.61)	36.20 (1.76)	33.66 (1.64)	0.39 [−0.38, 1.16]	0.16 [−0.53, 0.86]	*p* = 0.69	*d* = 0.16

*n*, sample size; MRT.h, Metabolic Equivalent of Task.

^4^Change scores represent the mean change from pre-test to post-test with 95% confidence intervals.

^5^Effect sizes (Cohen’s *d*) are calculated as difference in means between post-test and pre-test divided by standard deviation at pre-test.

^6^*p*-values represent the statistical significance of between-group differences in change scores derived from the linear mixed model analysis.

Inferential statistics revealed no baseline (pre-intervention) or follow-up (post-intervention) group differences in activity parameters. When examined together, the results of the linear mixed model analysis of pre-post change scores did not identify statistically significant between-group differences in ActivPAL™ metrics. Figure 4 illustrates these results. While no statistically significant between-group differences were identified, the direction of change for several outcomes, including daily step count, stepping duration, and sit/lie duration, favored the dance group. Effect sizes (Cohen’s d) were calculated as within-group pre-post effect sizes, derived from the difference between post-test and pre-test means divided by the baseline standard deviation. Effect sizes ranged from moderate-to-large (sit/lie duration, *d* = 0.52; stepping duration, *d* = 0.72; daily step count, *d* = 0.91) to small (standing duration, *d* = 0.17), (sit-to-stand transitions, *d* = 0.35; stand-to-sit transitions, *d* = 0.44; and energy expenditure, *d* = 0.16).

**Figure 4:**
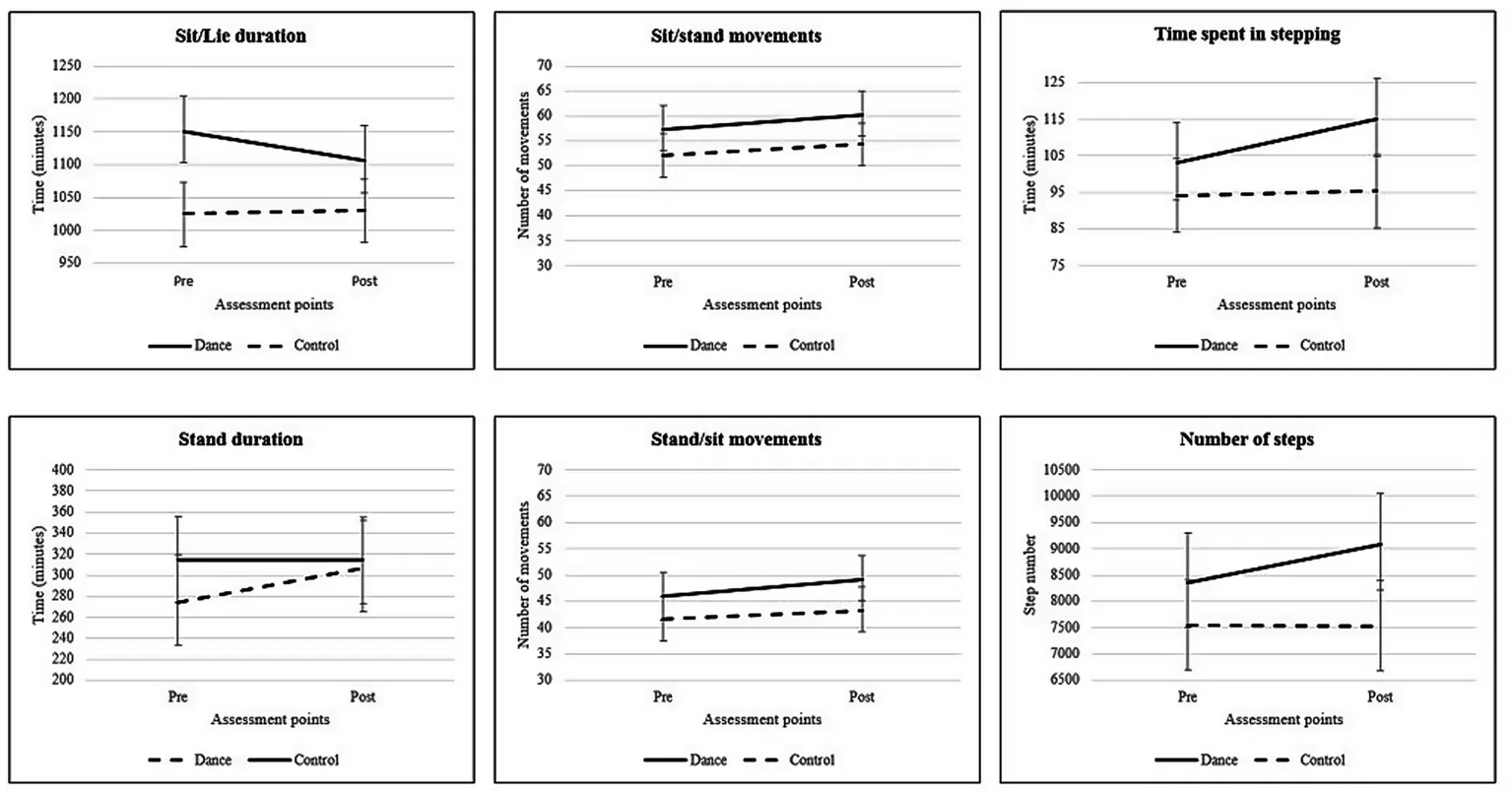
Level of activity measured using ActivPAL™ activity monitors: Dance (*n* = 16) vs. Control (*n* = 16) group, before and after the 3 months dance intervention.

## 4 Discussion

To our knowledge, this is the first study to objectively evaluate habitual physical activity following a dance for Parkinson’s disease intervention using ActivPAL™ devices. Contrary to our prediction, results related to the volume of daily activity exhibited no significant changes in daily step count, stepping duration, sit/lie duration, standing duration, sit-to-stand transitions, stand-to-sit transitions, or energy expenditure between the dance and control groups. Although statistically significant between-group differences were not detected, moderate-to-large effect sizes were observed for stepping duration and daily step count. However, these findings should be interpreted cautiously given the small sample size and absence of statistically significant effects. As the sample size calculation for the parent study was based on dual-task Timed Up and Go performance rather than habitual physical activity outcomes, the present analysis may have been underpowered to detect statistically significant differences in ActivPAL™ measures. Consequently, effect sizes were also considered to assist interpretation of the magnitude of observed within-group changes. These findings suggest outcomes that may warrant further investigation in a fully powered study.

We expected that the dance group would show a statistically significant improvement in habitual physical activity compared to controls. In the same cohort of participants, we previously reported that dance participation significantly improved functional mobility and motor symptoms (Haputhanthirige et al., 2023; Kalyani et al., 2020). Such gains were expected to flow through to post-program habitual physical activity, for example, by boosting physical capability. The failure to find support for our hypothesis suggests that physical capability improvements under structured or supervised conditions do not necessarily translate into changes in habitual activity outside those environments (Holleran et al., 2023). Consequently, for interventions to have sustainable benefits on habitual physical activity, they may need to incorporate additional behavioral strategies, goal setting, self-monitoring, or community engagement components alongside physical capability training. Adjusting outcomes to include a mix of capability as well as a wider range of habitual activity measures (e.g., activity bout duration, activity accumulation patterns, and temporal distributions of activity across the day), is also suggested for future research.

A second possible explanation for the lack of statistically significant findings for post program habitual physical activity was the relatively high baseline habitual physical activity level of participants in both groups. The dance group accumulated an average of 8,354 steps/day and the control group 7,554 steps/day at baseline. A study examining habitual daily activity suggested that approximately 7,000–10,000 steps/day may be considered sufficient for healthy older adults (Tudor-Locke et al., 2011). Consequently, participants may have had limited capacity to substantially increase their habitual daily physical activity, creating a potential ceiling effect. Similar challenges have been reported in exercise intervention studies where participants are already physically active at study entry (Schootemeijer et al., 2020; Mediano et al., 2018), potentially representing a selection bias. Another possibility is that the improvements in gait, dual-task gait performance, cognition, functional mobility, and other outcomes previously reported from this cohort may require a longer period to translate into measurable changes in habitual physical activity behavior (El Hayek et al., 2023). Future studies could implement an inclusion criterion targeting people below a specified activity threshold.

Several limitations should be considered when interpreting these findings. Obtaining stable estimates of habitual physical activity in people with PD can be challenging because of factors such as fluctuations in motor and non-motor symptoms, despite stable medication regimens (Jankovic, 2005). These fluctuations may have influenced activity levels during the monitoring periods. Furthermore, daily physical activity is affected by numerous factors including social participation, leisure pursuits, environmental conditions, and day-to-day variation in routine, and there is the possibility that we captured an atypical or reduced habitual activity week, after program completion. As habitual physical activity was measured over only five consecutive days at each time point, a stable estimate may not have been captured. This risk could have been compounded by the placement of the ActivPAL, which meant we could not distinguish between sitting and lying and may have underestimated activity. The possibility that our findings were affected by unstable habitual physical activity measurement could be addressed through replication studies using longer pre- and post-intervention monitoring periods, together with activity diaries to identify unusual activity demands, acute illness, or symptom fluctuations, and different device placement. An additional limitation was the unequal gender distribution between groups at baseline. Although gender was included as a covariate in all analyses, the possibility of residual confounding cannot be excluded, particularly given the relatively small sample size. Furthermore, the parent study was powered based on dual-task Timed Up and Go outcomes rather than habitual physical activity outcomes, and therefore may have been underpowered to detect statistically significant differences in ActivPAL™ measures. Future trials should undertake an *a priori* sample-size calculation based specifically on the selected objectively measured habitual physical activity outcome and an appropriately justified expected between-group difference. Furthermore, including a larger sample with true randomization would provide more robust estimates of habitual physical activity and post-program effects.

Taken together, the findings suggest that although dance for Parkinson’s disease classes improved some outcomes as previously reported, these improvements did not translate into increased habitual physical activity following program completion. Despite this finding, there are some promising research leads from this research. For example, the moderate-to-large effect sizes observed for stepping duration and daily step count in dance participation are clearly outcomes that warrant further attention in a fully powered study. Importantly, this study shows the feasibility of measuring habitual physical activity before and after program participation, and the methodological suggestions can shape the future studies we need to better understand how to leverage improvements from structured programs into meaningful changes in habitual physical activity for people with PD.

## Data Availability

The datasets generated and/or analysed during the current study are available from the corresponding author upon reasonable request.
